# Early progression of brain atrophy in patients with anti-N-methyl-D-aspartate receptor encephalitis: Case reports

**DOI:** 10.1097/MD.0000000000006776

**Published:** 2017-04-28

**Authors:** Hiroshi Kataoka, Nobuhiro Sawa, Yasuyo Tonomura, Satoshi Ueno

**Affiliations:** Department of Neurology, Nara Medical University, Kashihara, Nara, Japan.

**Keywords:** brain atrophy, cerebral atrophy, encephalitis, NMDA receptor, NMDAR encephalitis

## Abstract

**Background:**

Anti-N-methyl-D-aspartate receptor (anti-NMDAR) encephalitis responds to immunnotherapy, and approximately 80% of patients with this disorder fully recover or have only minor sequelae. Brain magnetic resonance imaging (MRI) does not show a specific abnormality, but some patients have progressive cerebral atrophy. The cerebral atrophy can become reversible after clinical improvement.

**Methods:**

We describe 3 patients with diffuse cerebral atrophy (DCA) on serial brain MRI.

**Results:**

Two women had the typical spectrum of this disorder, and one man had mainly psychiatric symptoms. In a woman with an ovarian tumor, DCA was reversible and DCA developed within about a half month. In another woman without a tumor, DCA was evident within 19 days and had progressed over the course of 4 years. The titers of anti-NMDAR antibodies in serum and cerebrospinal fluid (CSF) initially decreased, and low titers of the antibodies persisted. In a man without a tumor, DCA progressed within 14 days, and during this short period, he did not receive prolonged treatment with corticosteroids, various antiepileptic agents, or propofol, and he was free of seizures and ventilatory support.

**Conclusion:**

Not only a woman but also a man with anti-NMDAR encephalitis can have DCA in the early phase of this disorder. However, DCA can be reversible after clinical improvements. The early progression of DCA is not necessarily a poor prognostic factor.

## Abstract 2

Supplemental Digital Content is available in the text

## 1 Introduction

Anti-N-methyl-D-aspartate receptor (anti-NMDAR) encephalitis has been well recognized internationally. This disease is typically characterized by acute behavioral changes, prominent psychiatric symptoms, seizures, involuntary movements, autonomic instability, and central hypoventilation, and is associated with ovarian teratoma.^[1]^ Many patients respond to immune treatment, and approximately 80% of patients with this disorder fully recover or have only minor sequelae.^[1–3]^ Brain magnetic resonance imaging (MRI) does not show a specific abnormality in many patients with anti-NMDAR encephalitis,^[1–3]^ but some patients have progressive cerebral atrophy.^[2–4]^ Recent longitudinal studies have shown that cerebral atrophy can become reversible after clinical improvement,^[4,5]^ and cerebellar atrophy is reported to be a poor prognostic factor.^[5]^ We describe 3 patients with diffuse cerebral atrophy (DCA) on serial brain MRI.

## 2 Case reports

### 2.1 Case 1

**Figure 1:**
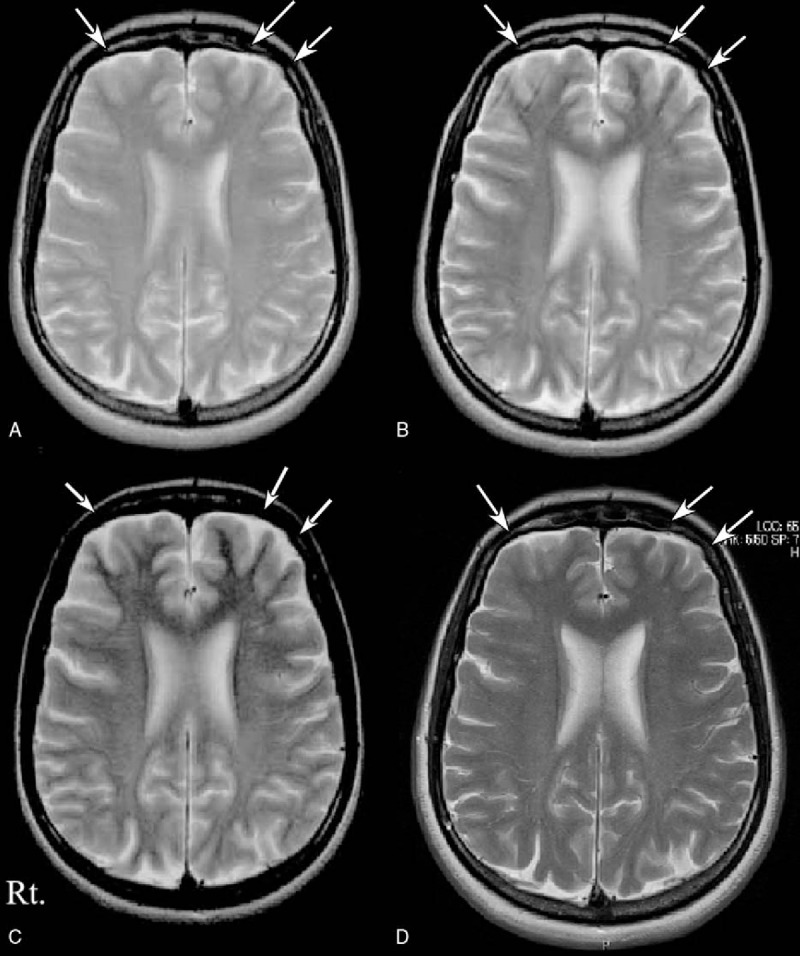
Serial MRI studies in Patient 1 (upper panels). A second brain MRI obtained 16 days after admission (panel B) showed the development of diffuse brain atrophy as compared with first brain MRI obtained on admission (panel A). Brain MRI obtained 6 months after admission showed the further progression of diffuse brain atrophy (panel C). Fourteen years after admission, the diffuse brain atrophy had partially improved (panel D).

A 29-year-old previously healthy woman had psychiatric symptoms, central hypoventilation, seizures, involuntary movements, and autonomic instability (see ^[6]^ for details). In brief, inappropriate behavior and impaired episodic memory were presented, and subsequently, she was confused, leading to be admitted to our hospital in January 2002. Twelve days after the admission, the consciousness level was dropped with generalized seizures and involuntary movements. She was given anticonvulsant medications. On day 16 after admission, a second brain MRI showed DCA with no abnormal intensity as compared with the first brain MRI, performed on day 1 (Fig. 1A, B). On day 24, she received mechanical ventilation, since respiratory failure developed. Intravenous sedation was begun because of frequent generalized seizures. She was treated with intravenous dexamethasone (16 mg/day) and immunoglobulin (5 g/day, 3 times), but hypothermia, hypersalivation, and cardiac arrhythmias occurred. The level of consciousness increased and ventilatory support was withdrawn on day 68. Six months after admission, brain MRI showed the further progression of diffuse brain atrophy (Fig. 1C). In September, she received an ovarian cystectomy and bilateral ovarian tumors were removed. Bilateral mature cystic teratoma was histopathologically confirmed. Twenty-five months after admission, she was able to return to work. Fourteen years after admission, the severity of DCA had partially decreased (Fig. 1D).

### 2.2 Case 2

**Figure 2:**
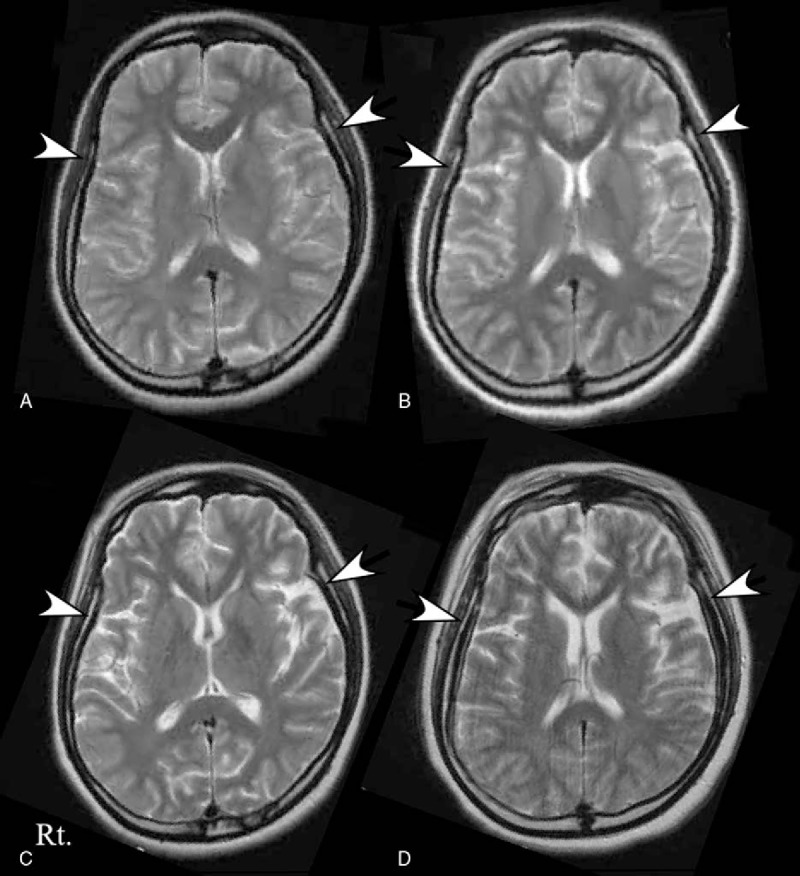
Serial MRI studies in Patient 2. The first brain MRI was normal (panel A). The second brain MRI, obtained on day 19, showed the presence of diffuse brain atrophy (panel B, white arrows). The diffuse brain atrophy showed progression on the third brain MRI, obtained on day 42 (panel C), and the final follow-up MRI, obtained 3 years 8 months after admission (panel D).

Psychiatric symptoms developed in a previously healthy 46-year-old woman, and she received prolonged ventilatory support because of central hypoventilation, seizures, involuntary movements, and autonomic instability (see ^[7]^ for details). In brief, in late March 2008, she presented with distortion of visual perception and unusual behavior. In early April, she was entered to our hospital because of generalized seizures and delusional thinking. Brain MRI on day 1 was normal (Fig. 2A). Twelve days after admission, involuntary movements and frequent seizures developed. She required ventilatory support and intravenous sedative drugs. On day 19 and day 42 after admission, brain MRI showed that DCA was progressing without abnormal intensity (Fig. 2B, C). The disease course is shown in a previously published figure (Supplemental Figure 1 see ^[7]^ for details). Autonomic instability also occurred. The involuntary movements and seizures were unresponsive to antiepileptic drugs. High doses of intravenous propofol (4 mg/kg/h) and midazolam (3.75 mg/kg/h) werr needed while she received mechanical ventilation from April 2008 through February 2011. She repeatedly received intravenous steroids (500 mg/day, 3 days, 2 times), plasmapheresis (4 times, alternating days), and intravenous immunoglobulin (0.4 g/kg/day, 5 days). A teratoma or other type of tumor was not found. Frequencies of seizures and involuntary movements were decreased in November 2009, and the dose of intravenous propofol was slowly tapered. She was free of ventilatory support in February 2011. Brain MRI in December 2011 showed the further progression of DCA (Fig. 2D). In April 2012, she required constant nursing care, and she was bedridden and incontinent. Anti-NMDAR antibody titers in serum and cerebrospinal fluid (CSF) samples were 1:1600 and 1:320, respectively, at the first sampling and 1:200 and 1:80 4 years after the onset of disease.

### 2.3 Case 3

**Figure 3:**
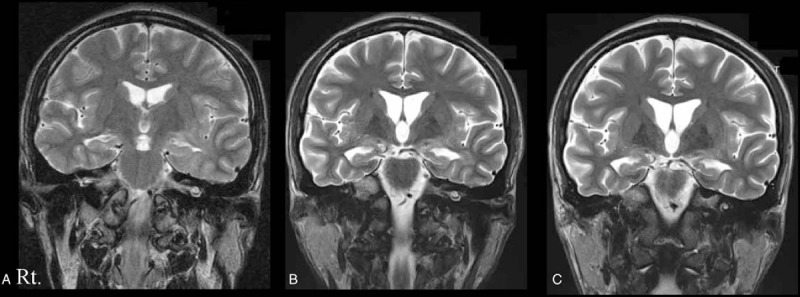
Serial MRI studies in Patient 3. The first brain MRI on day 1 was normal (panel A). Diffuse brain atrophy was evident on the second brain MRI, obtained on day 14 (panel B). Brain atrophy was evident on the final follow-up MRI, obtained 64 days after admission (panel C).

A 18-year-old previously healthy man presented with pyrexia and headache in late February 2012. He was admitted to another hospital for a diagnosis of viral meningitis because the CSF showed 251 lymphocytes, a protein concentration of 79 mg/dL, and a glucose concentration of 55 mg/dL. In early March, psychiatric symptoms developed, and he was transferred to our hospital. He had irritability, psychiatric symptoms with hallucinations, and impaired orientation. Meningismus was evident. The results of brain MRI were normal (Fig. 3A). Steroid pulse therapy (1 g, 3 days) was begun, and he received intravenous acyclovir (500 mg, three times daily) for a presumptive diagnosis of herpes simplex encephalitis. Intravenous midazolam (maximum dosage 0.5 mg/kg/h) was needed because of the development of psychiatric symptoms. Catatonia in both upper limbs was evident. On day 6 after admission, repeated steroid pulse therapy (1 g, 3 days) was begun, and the severity of disease decreased. On day 14, a second brain MRI showed the presence of DCA (Fig. 3B). On day 20, the patient was admitted to an intensive care unit because of hypovolemic shock due to massive hemorrhagic diarrhea for 4 days and disseminated intravascular coagulation. After admission to the intensive care unit, he was given dexmedetomidine (10 μg/kg/h), midazolam (10 mg, 21 times), diazepam (10 mg, 4 times), or risperidone (1 mg, dairy) because psychiatric symptoms often occurred. Additional immunotherapy was not performed because of severe infections enteritis. Two months after admission, he could have a brief conversation. On day 64, DCA persisted (Fig. 3C). Three months after admission, he had complete recovery, and no tumor was found.

All patients were given a diagnosis of anti-NMDAR encephalitis on immunocytochemical studies.^[1,8]^ In Patients 1 and 3, the diagnosis was made after clinical improvement. Oral informed consent was obtained from the patients as well as from their families.

## 3 Discussion

In our patients, DCA developed in patient 1 within about a half month. In Patients 2 and 3, DCA progressed within 19 and 14 days, respectively. We found that DCA occurred in the early stage of anti-NMDAR encephalitis. A recent longitudinal study reported that DCA developed within 1 to 2 months after symptom onset in 5 of 15 patients.^[5]^ The authors mentioned that the mechanism of DCA was unknown; however, they speculated that potential risk factors included systemic complications, status epilepticus, malnutrition, prolonged use of corticosteroids, long-term exposure to various antiepileptic agents, and prolonged use of propofol. In particular, brain atrophy is likely to develop in patients with status epilepticus persisting for longer than 24 hours after the onset of anesthetic therapy, as seen in Patient 2.^[9]^ Hypoxia due to systemic conditions or prolonged ventilatory support might also contribute to the development of brain atrophy.^[10]^ DCA in our 2 women might have been associated with these multifactorial risks. In our male patient, DCA progressed within 14 days, and during this short period, he did not receive prolonged treatment with corticosteroids, various antiepileptic agents, or propofol, and he was free of seizures and ventilatory support. He received high-dose corticosteroids, which can induce reversible short-term loss of brain volume,^[11]^ but the brain atrophy of the patients was persisted. Thus, anti-NMDAR encephalitis itself is also likely to contribute to the cerebral atrophy. Previously, because of the association of NMDAR antagonists with the development of brain atrophy, the mechanism of brain atrophy was attempted to be explained on the basis of a direct role of antibodies on NMDAR internalization. However, other authors have taken a somewhat negative view of this speculation on the basis of in vitro and in vivo experiments with NMDAR antibodies.^[10]^ In our patients, brain atrophy predominantly involved the frontotemporal regions, as reported previously.^[4]^ This area includes a high density of NMDA receptor, and the binding epitope of the antibody is a part of the NR1-subunit of the NMDAR on postsynaptic dendrites in the forebrain and hippocampus.^[1,2]^ These findings might suggest an immunological cause of the brain atrophy.

Unique findings in our study were that DCA was seen not only in 2 women but also in a man. Previously, all women with DCA showed a typical spectrum of anti-NMDAR encephalitis.^[4,5,12]^ Our 2 women had the typical spectrum of this disorder; on the contrary, the man had mainly psychiatric symptoms. Second, despite the progressive DCA, a gradual improvement in anti-NMDAR encephalitis was evident clinically, consistent with the findings of a previous study.^[5]^ In Patient 2, who had DCA that had progressed over the course of 4 years, the titers of anti-NMDAR antibodies in serum and CSF initially decreased, and low titers of the antibodies persisted. In a previous study of 2 patients with cerebellar atrophy, the titer of CSF antibodies was mildly increased in 1 patient, whereas CSF antibodies were no longer detectable in another patient.^[5]^ The titer of anti-NMDAR antibodies is not likely to correlate with brain atrophy; however, this must be confirmed in a further longitudinal study. Third, DCA became partially reversible in Patient 1. Such reversible brain atrophy has been described previously.^[4,5]^ This finding was seen in 33% of patients with anti-NMDAR encephalitis, and reversible DCA was seen in patients with good long-term outcomes.^[4,5]^ In contrast, DCA was not reversible in Patient 2, most likely because this patient was not given second-line treatment at the family's request. Previously, patients with DCA were not given second-line immunotherapy, but the reversal of DCA was accelerated by first-line immunotherapy in 1 patient.^[5]^

## 4 Conclusions

Our experience indicates that not only a woman but also a man with anti-NMDAR encephalitis can have DCA in the early phase of this disorder. However, DCA can be reversible after clinical improvements. The early progression of DCA is not necessarily a poor prognostic factor.

## Supplementary Material

Supplemental Digital Content
